# A scorpion venom peptide derivative BmKn‒22 with potent antibiofilm activity against *Pseudomonas aeruginosa*

**DOI:** 10.1371/journal.pone.0218479

**Published:** 2019-06-14

**Authors:** Kittitat Teerapo, Sittiruk Roytrakul, Anchalee Sistayanarain, Duangkamol Kunthalert

**Affiliations:** 1 Department of Microbiology and Parasitology, Faculty of Medical Science, Naresuan University, Phitsanulok, Thailand; 2 Genome Institute, National Center for Genetic Engineering and Biotechnology, National Science and Technology Development Agency, Thailand Science Park, Pathumthani, Thailand; 3 Centre of Excellence in Medical Biotechnology, Faculty of Medical Science, Naresuan University, Phitsanulok, Thailand; Nanyang Technological University, SINGAPORE

## Abstract

*Pseudomonas aeruginosa* is a leading cause of nosocomial and serious life-threatening infections and infections caused by this bacterium continue to pose a major medical challenge worldwide. The ability of *P*. *aeruginosa* to produce multiple virulence factors and in particular to form biofilms makes this bacterium resistant to all known antibiotics. As a consequence, standard antibiotic therapy are increasingly become ineffective to clear such infections associated with biofilms. In search for novel effective agents to combat *P*. *aeruginosa* biofilm infections, a series of the BmKn‒2 scorpion venom peptide and its truncated derivatives were synthesized and their antibiofilm activities assessed. Among the peptides tested, BmKn‒22 peptide, which was a modified peptide of the parental BmKn‒2 scorpion venom peptide, clearly demonstrated the most potential inhibitory activity against *P*. *aeruginosa* biofilms without affecting the bacterial growth. This peptide was not only capable of inhibiting the formation of *P*. *aeruginosa* biofilms, but also disrupting the established biofilms of *P*. *aeruginosa*. Additionally, BmKn‒22 peptide was able to inhibit the production of key virulence factor pyocyanin of *P*. *aeruginosa*. Our results also showed that BmKn‒22 peptide significantly reduced *lasI* and *rhlR* expression, and suggested that BmKn‒22 peptide-mediated inhibition of *P*. *aeruginosa* biofilms and virulence factors was achieved through the components of quorum-sensing systems. Combination of BmKn‒22 peptide with azithromycin resulted in a remarkable reduction *P*. *aeruginosa* biofilms. Since this peptide exhibited low toxicity to mammalian cells, all our results therefore indicate that the BmKn‒22 peptide is a promising antibiofilm agent against *P*. *aeruginosa* and warrant further development of this peptide as a novel therapeutic for treatment of *P*. *aeruginosa*‒associated biofilm infections.

## Introduction

*Pseudomonas aeruginosa* is the most common opportunistic Gram-negative bacterium that has become a leading cause of nosocomial and serious life-threatening infections, especially in patients with compromised host defense mechanisms. *P*. *aeruginosa* can form biofilms and produces multiple virulence factors that are implicated in pathogenesis of infections. Biofilms are a densely packed community of bacterial cells that attach on surfaces and that are embedded in a self-produced extracellular polymeric substance [1, 2]. Bacterial cells grown in biofilms exhibit different physiology and phenotype from their planktonic counterpart [3, 4], and are more tolerant toward antibiotics and host immune-mediated clearance than the same organism growing planktonically [5]. Several studies documented that the bacteria on biofilm structures exhibited up to 1000-fold increased resistance to a wide range of antimicrobial agents [6, 7]. The ability of *P*. *aeruginosa* to form biofilms thus makes this bacterium recalcitrant to a large number of the currently available antibiotics. High incidence of multidrug resistance in *P*. *aeruginosa* has extensively been reported and this continues to be increasing each year [8]. Accordingly, conventional antibiotic therapy is often insufficient to clear biofilm infections. Higher concentrations of antibiotics and/or combinations have been suggested to treat biofilm-related infections [9, 10]. However, excessive or improper uses of antibiotics have dramatically supported the development of bacterial resistant strains [11], leading to even greater difficulties in disease treatment. Biofilm-associated infections currently pose a major medical challenge globally. Therefore, the discovery and development of novel effective agents to combat biofilm-associated infections are extremely important. The World Health Organization (WHO) has recently announced that *P*. *aeruginosa* is one of the critical priority pathogens for which new antibiotics are urgently needed [12].

With this perspective, an innovative approach could be the development of antibiofilm agents with new modes of action that are different from those of currently used antibiotics [13]. Antimicrobial peptides (AMPs) are evolutionary conserved molecules founded in a wide range of organisms, and considered to play important roles in host innate immunity of all species [14, 15]. AMPs have attracted great attention as a novel class of antibiotics due to their prospective potency, rapid action and broad-spectrum antimicrobial activity against an array of microbes, including bacteria, viruses, fungi and protozoa [16]. Low potential of AMPs to induce bacterial resistance is also of significant feature [14, 16, 17], making these molecules more attractive for combating multidrug resistant bacteria.

BmKn‒2 is a basic, alpha-helical antimicrobial peptide that was derived from the venom of scorpion *Mesobuthus martensii* Karsch [18]. BmKn‒2 peptide has strong antimicrobial activity against both Gram-positive and Gram-negative bacteria including *Staphylococcus aureus*, *Micrococcus luteus*, *Bacillus subtilis*, *Escherichia coli*, *P*. *aeruginosa* [18] and *Neisseria gonorrhoeae* [19]. BmKn‒2 peptide also exerts anti-cancer activity against oral and colon cancer cells [20, 21]. Nevertheless, to the best of our knowledge, experimental data regarding the antibiofilm activity of this peptide against *P*. *aeruginosa* has not yet been reported. The present study therefore evaluated antibiofilm activity of the BmKn‒2 peptide as well as its derivatives against *P*. *aeruginosa* biofilms. The possible mechanism responsible for its activity was also investigated.

## Materials and methods

### Peptides

BmKn‒2 peptide and its truncated derivatives were synthesized by ChinaPeptides Co., Ltd. (Shanghai, China) or GenScript (Piscataway, USA); the purity of peptides was > 90%. The amino acid sequence and physico-chemical properties of the studied peptides are presented in Table 1. Their molecular weight, net charge and % hydrophobicity were calculated using APD3: Antimicrobial Peptide Calculator and Predictor [22] whereas helix and secondary structures were predicted by NPS@: network protein sequence analysis [23]. BmKn‒2 peptide and its derivatives were dissolved in their vehicle, dimethyl sulfoxide (DMSO; ≥ 99.5%, Sigma, France) and further diluted in culture medium to obtain desired concentrations.

**Table 1:** Amino acid sequence and predicted physico-chemical properties of BmKn-2 peptide and its derivatives.

Peptides	Amino acid sequence	Molecular weight (Da)	Length	Net charge	Hydrophobicity (%)	Helix	Structure prediction
**BmKn-2**	FIGAIARLLSKIF	1448.81	13	+2	56.23	76.92	CHHHHHHHHHHCC
**BmKn-21**	FIGAIARLLSKI	1301.64	12	+2	66.67	75	CHHHHHHHHHCC
**BmKn-22**	FIGAIARLLSK	1188.48	11	+2	48.64	63.64	CCHHHHHHHCC
**BmKn-23**	FIGAIARLLS	1060.3	10	+1	55.8	70	CHHHHHHHCC
**BmKn-24**	FIGAIARLL	973.23	9	+1	62.56	66.67	CHHHHHHCC
**BmKn-25**	FIGAIARL	860.07	8	+1	57.88	0	CCCCCCCC
**BmKn-26**	FIGAIAR	746.91	7	+1	51.86	0	CCCCCCC

### Bacterial strain and growth condition

*P*. *aeruginosa* PAO1 was obtained from the Spanish Type Culture Collection (CECT, Valencia, Spain). This bacterial strain was frozen and kept at ‒ 80°C. Prior to each experiment, two subcultures were prepared on Luria-Bertani (LB) agar (BD Difco™, Le Pont de Claix, France) and incubated under aerobic condition at 37 ^O^C for 24 h. A single colony was then taken and bacterial suspensions were freshly prepared in LB broth for subsequent experiments.

### Biofilm susceptibility assay

The effect of BmKn‒2 peptide and derivatives on biofilm formation of *P*. *aeruginosa* PAO1 was determined according to the method published previously [24, 25] with some modifications. Briefly, 100 μL of the test peptides was plated into the wells of flat-bottomed 96-well microtiter plates (Nunc™, Roskilde, Denmark) at the final concentrations of 200 ‒ 800 μM. Aliquots of the *P*. *aeruginosa* PAO1 suspension were then inoculated to the wells of 96-well microtiter plates to obtain a final concentration of 10^6^ CFU/mL. Culture without the test peptides was used as the untreated control. After incubation at 37 ^O^C for 24 h without agitation, planktonic cells were removed and the plates were gently rinsed twice with phosphate buffered saline (PBS) pH 7.4. Biofilms were then stained with 0.1% crystal violet solution (Merck, Darmstadt, Germany) for 10 minutes at room temperature. Excess stains were rinsed off with PBS pH 7.4 and the plates were left dry at 37 ^O^C for 2 h. Subsequently, biofilm biomass was solubilized with 30% acetic acid and the optical density (OD) measured at 550 nm using a microplate reader (BioTek Synergy HT).

Antibiofilm activity of the test peptides was also examined against established biofilms as follows. Aliquots of the *P*. *aeruginosa* PAO1 suspension adjusted to a final concentration of 10^6^ CFU/mL were seeded into the wells of flat-bottomed 96-well microtiter plates (Nunc). Biofilms were established at 37°C for 24 h. After incubation, nonadherent planktonic cells were removed by gently washing the wells with sterile PBS pH 7.4. Preformed biofilms were then treated with the test peptides at concentrations of 200 ‒ 800 μM and incubated at 37°C for 24 h. Culture without the test peptides served as the untreated control. After the incubation, the test peptides were aspirated gently and the plates were rinsed twice with PBS pH 7.4. Biofilm biomass was subsequently quantified by 0.1% crystal violet staining as previously described.

### Growth assay

The effects of BmKn‒2 peptide and its derivatives on growth of *P*. *aeruginosa* PAO1 were carried out as described previously [25] with some modifications. Bacterial suspension (10^6^ CFU/mL) and the test peptides (800 μM, final concentration) were incubated at 37 ^O^C at 150 rpm. Bacterial culture without the test peptides was used as the untreated control. After 24 h, bacterial concentration was evaluated. Cultures from each treatment were taken and 10-fold serial dilution was performed; 100 μL of each dilution was then spread on LB agar plates. Following incubation at 37°C for 24 h, the colonies were counted and expressed as log_10_ CFU/mL.

### Pyocyanin assay

Pyocyanin pigments produced by *P*. *aeruginosa* PAO1 after exposure to the BmKn‒22 peptide were determined according to the protocol described previously [26]. Briefly, 750 μL of *P*. *aeruginosa* suspension (10^7^ CFU/mL, final concentration) was mixed with 250 μL of LB broth containing the test peptide at final concentrations of 200 ‒ 800 μM and incubated at 37 ^O^C for 24 hours with agitation (150 rpm). Control culture without the test peptide was simultaneously propagated. After centrifugation at 4,500 rpm for 10 minutes, supernatant was collected and pyocyanin was extracted with chloroform followed by 0.2 N HCl. The suspension was centrifuged once at 4,500 rpm for 10 minutes, and the pink phase layer was subjected to optical density determination at 380 nm using a microplate reader (BioTek Synergy HT).

### Quantitative real-time polymerase chain reaction (qRT-PCR)

The effects of BmKn‒22 peptide on the expression of quorum sensing‒related genes in *P*. *aeruginosa* PAO1 were assessed by qPCR. Briefly, bacterial suspension at approximately 10^7^ CFU/mL was cultured in the presence or absence of the test peptide (800 μM, final concentration) at 37 ^O^C for 10 h using a shaking incubator. After centrifugation at 4,500 rpm for 10 minutes, the bacterial cells were harvested and subsequently subjected to total RNA extraction using TRIzol^®^ reagent (Invitrogen, USA) as per the manufacturer’s instruction. The concentration of the extracted RNA was measured using Nanodrop spectrophotometer (NanoDrop Technologies, USA). Thereafter, 1 μg of extracted RNA was reverse-transcribed into cDNA using Random Hexamer primer and RevertAid First Strand cDNA synthesis kit (Fermantas), which was amplified by real-time PCR. The primers for the genes *lasI*, *lasR*, *RhlI*, *RhlR* and *16S rRNA* were used (Table 2). All primers were synthesized by the Integrated DNA Technology (IDT), Canada. The reaction mixture consisted of 1× AccuPower^®^ 2X GreenStar*™* qPCR Master Mix, 0.4 μmol/L each forward primer and reverse primer and 1 μL of cDNA in a final volume of 10 μL. Thermal cycles used was 95 ^O^C for 2.5 min, followed by 40 cycles of 95 ^O^C for 30 sec, 60 ^O^C for 30 sec and 72 ^O^C for 30 sec. The expression level of the target gene was normalized to 16*S rRNA* gene. Relative expression of the target genes was obtained from 2^‒ΔΔct^.

**Table 2:** List of primers used in this study.

Target gene	Primer sequence 5’ to 3’	Reference
***lasI***	**F:** CTACAGCCTGCAGAACGACA	[27]
	**R:** ATCTGGGTCTTGGCATTGAG	
***lasR***	**F:** ACGCTCAAGTGGAAAATTGG	[27]
	**R:** GTAGATGGACGGTTCCCAGA	
***rhlI***	F: CTC TCTGAATCGCTGGAAGG	[27]
	R: GACGTCCTTGAGCAGGTAGG	
***rhlR***	F: AGGAATGACGGAGGCTTTTT	[27]
	R: CCCGTAGTTCTGCATCTGGT	
***16S rRNA***	F: CGTCCGGAAACGGCCGCT	[28]
	R: CTCTCAGACCAGTTACGG	

### Determination of minimum inhibitory concentration (MIC)

MIC of azithromycin against *P*. *aeruginosa* was determined by using broth microdilution assay according to the protocol previously described [29] with some modifications. Twofold serial dilutions of azithromycin (AZM; Sigma, St. Louis, USA) were prepared in LB broth in the wells of flat-bottomed 96-well microtiter plates (Nunc). Aliquots of *P*. *aeruginosa* PAO1 suspension at a final concentration of 10^6^ CFU/mL was added to the wells of flat-bottomed 96-well microtiter plates (Nunc). After incubation at 37 ^O^C for 24 h, the MIC value was examined which was defined as the minimum concentration in the first well that showed no visible growth.

### Antibiofilm activities of BmKn‒22 peptide in combination with azithromycin

Briefly, *P*. *aeruginosa* PAO1 suspension (10^6^ CFU/mL, final concentration) was added to the wells of flat-bottomed 96-well microtiter plates (Nunc) containing the test peptide, azithromycin (AZM; Sigma) alone or in combinations. The final concentrations of the test peptide ranged from 200 ‒ 800 μM, and the concentration of azithromycin was 64 μg/mL (1/2 MIC). Biofilm formation was employed by measuring biofilm biomass stained with crystal violet as a protocol described in previous section.

### Hemolytic assay

Hemolytic activity of the test peptides was assayed according to a protocol described previously [30]. Suspension of 2% sheep red blood cells (100 μL) prepared in PBS pH 7.4 was incubated with 100 μL of the test peptides (800 μM, final concentration). After incubation at 37 ^O^C for 1 h, the suspension was centrifuged at 1,000 g for 5 min followed by transferring 100 μL of the supernatant to 96 well-microtiter plate (Nunc). Released hemoglobin was then determined by measuring an absorbance at 405 nm using a microplate reader (BioTek Synergy HT). Positive and negative controls in this assay were 1% Triton X-100 and PBS pH 7.4, respectively. Hemolysis (%) was calculated using an equation: (OD405 nm peptide ‒ OD405 nm PBS pH 7.4)/(OD405 nm 1% Triton X-100 ‒ OD405 nm PBS pH 7.4) × 100.

### Cytotoxicity assay

The toxicity of the test peptides against L929 mouse fibroblast cells was assessed by 3-(4,5-dimethylthiazol-2-yl)-2,5-diphenyltetrazolium bromide (MTT) assay [31]. L929 mouse fibroblast cells from American Type Culture Collection (ATCC, Manassas, VA, USA) were cultured in Dulbecco's Modified Eagle Medium (DMEM; HyClone, Utah, USA) supplemented with 10% fetal bovine serum (Gibco, South America), 10 mM 4-(2-hydroxyethyl)-1 piperazineethanesulfonic acid (HEPES; HyClone, Utah, USA), 2 mM L-glutamine (Gibco, Brazil) and 100 U/mL penicillin and 100 μg/ml streptomycin (Gibco, USA) in a 5% CO_2_ atmosphere at 37°C. L929 cells (2×10^4^ cells/well) were seeded in 96-well plates (Nunc) and incubated overnight. The culture medium was discarded and replaced by 100 μL of fresh DMEM containing the test compounds (final concentration 800 μM). Cells without the test compounds served as untreated control. After 24 h incubation, 20 μL of MTT solution (5 mg/mL; Sigma, St. Louis, MO, USA) was added and incubated for 3 h. The culture supernatant was removed and 100 μL dimethyl sulfoxide was added to each well to dissolve the formazan crystals. The absorbance was measured at 540 nm on a microplate reader (BioTek Synergy HT). Percentage of cell viability was calculated according to the equation: (OD of treated cells/ OD of untreated cells) × 100.

### Statistical analysis

The results were obtained from independent experiments as indicated, and expressed as mean ± standard error of mean (SEM). Difference between test and control was analyzed by two-tailed student’s *t*-test using SPSS version 20 software (SPSS, Chicago, IL, USA). *P* < 0.05 was considered statistically significant unless otherwise specified.

## Results

### Antibiofilm activities of BmKn‒2 peptide and its derivatives against *P*. *aeruginosa*

**Fig 1:**
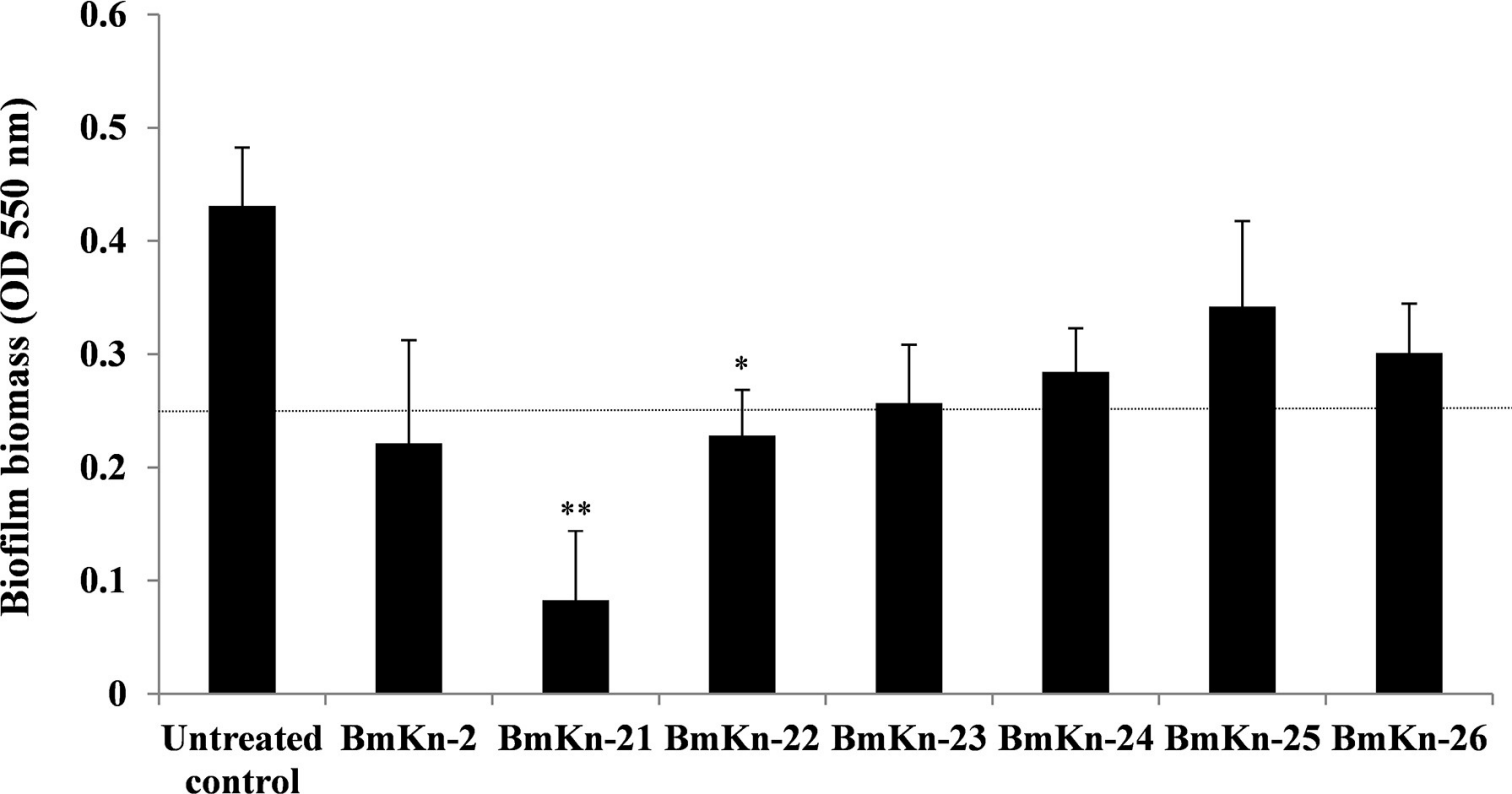
Antibiofilm activities of BmKn‒2 peptide and its derivatives against *P*. *aeruginosa*. Bacterial cells were incubated with BmKn‒2 peptide and derivatives at the final concentration of 800 μM. After incubation at 37°C for 24 h, biofilm biomass was assessed by crystal violet staining assay and OD measured at 550 nm. Data are expressed as mean ± SEM of three independent experiments. *, *P* < 0.05 and **, *P* < 0.01 compared with the untreated control. A dot black line represents an OD cut-off value.

BmKn‒2 peptide and its derivatives were initially assessed for their inhibitory activities against biofilms formed by *P*. *aeruginosa*, and the results are presented in Fig 1. It was found that biofilm biomass of *P*. *aeruginosa* was reduced when exposed to the test peptides, as compared with the untreated control. Although individual variabilities were observed, marked reduction of *P*. *aeruginosa* biofilms was obtained with the BmKn‒2, BmKn‒21, BmKn‒22 and BmKn‒23 peptides; significant antibiofilm activity observed with BmKn‒21 and BmKn‒22 peptides. Treatment with BmKn‒24, BmKn‒25 and BmKn‒26 peptides, however, produced less biofilm reduction activities.

### Effect of BmKn‒2 peptide and its derivatives on *P*. *aeruginosa* growth

**Fig 2:**
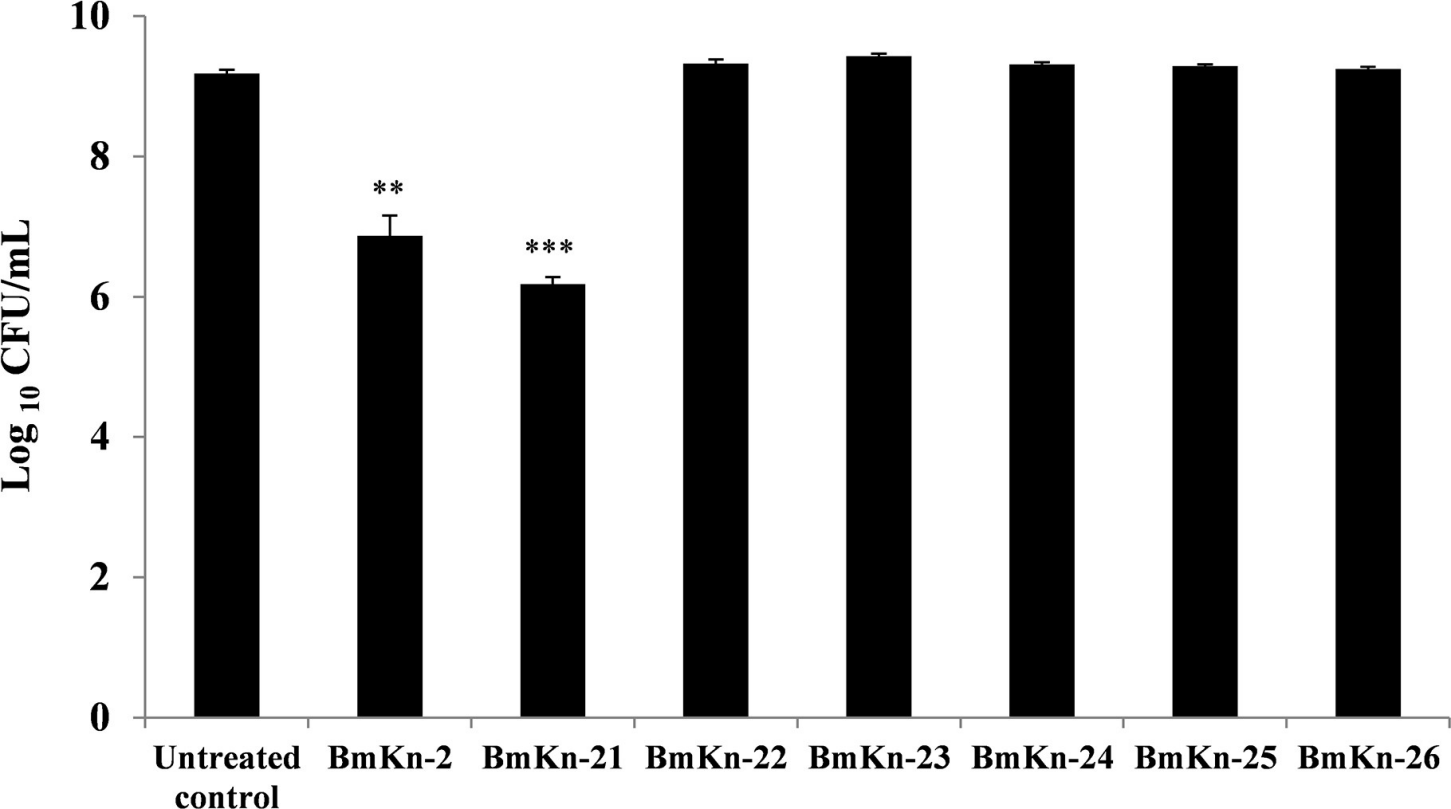
Effect of BmKn‒2 peptide and its derivatives on *P*. *aeruginosa* growth. Bacterial cells were treated with the test peptides and incubated at 37°C for 24 h. Bacterial growth was assessed by standard plate counts. Data are expressed as mean ± SEM of three independent experiments. **, *P* < 0.01 and ***, *P* < 0.001 compared with the untreated control.

To ensure that the observed reduction of *P*. *aeruginosa* biofilms by BmKn‒2 peptide and its derivatives was not caused by its growth inhibitory activity, growth of *P*. *aeruginosa* in the presence of the test peptides were quantified in terms of viable cell number. The results in Fig 2 showed the reduction in bacterial counts after incubation with BmKn‒2 and BmKn‒21 peptides. However, no significant (*P* > 0.05) differences in bacterial counts were observed with peptides BmKn‒22, BmKn‒23, BmKn‒24, BmKn‒25 and BmKn‒26, as compared with the untreated control. These results thus demonstrated that the BmKn-2 and its derivatives, except the BmKn‒2 and BmKn‒21 peptides, had no effects on growth of *P*. *aeruginosa* and suggested that biofilm reduction activities of such peptides were not due to growth inhibitory activities.

### Hemolytic activity and cytotoxicity of BmKn‒2 peptide and its derivatives

**Fig 3:**
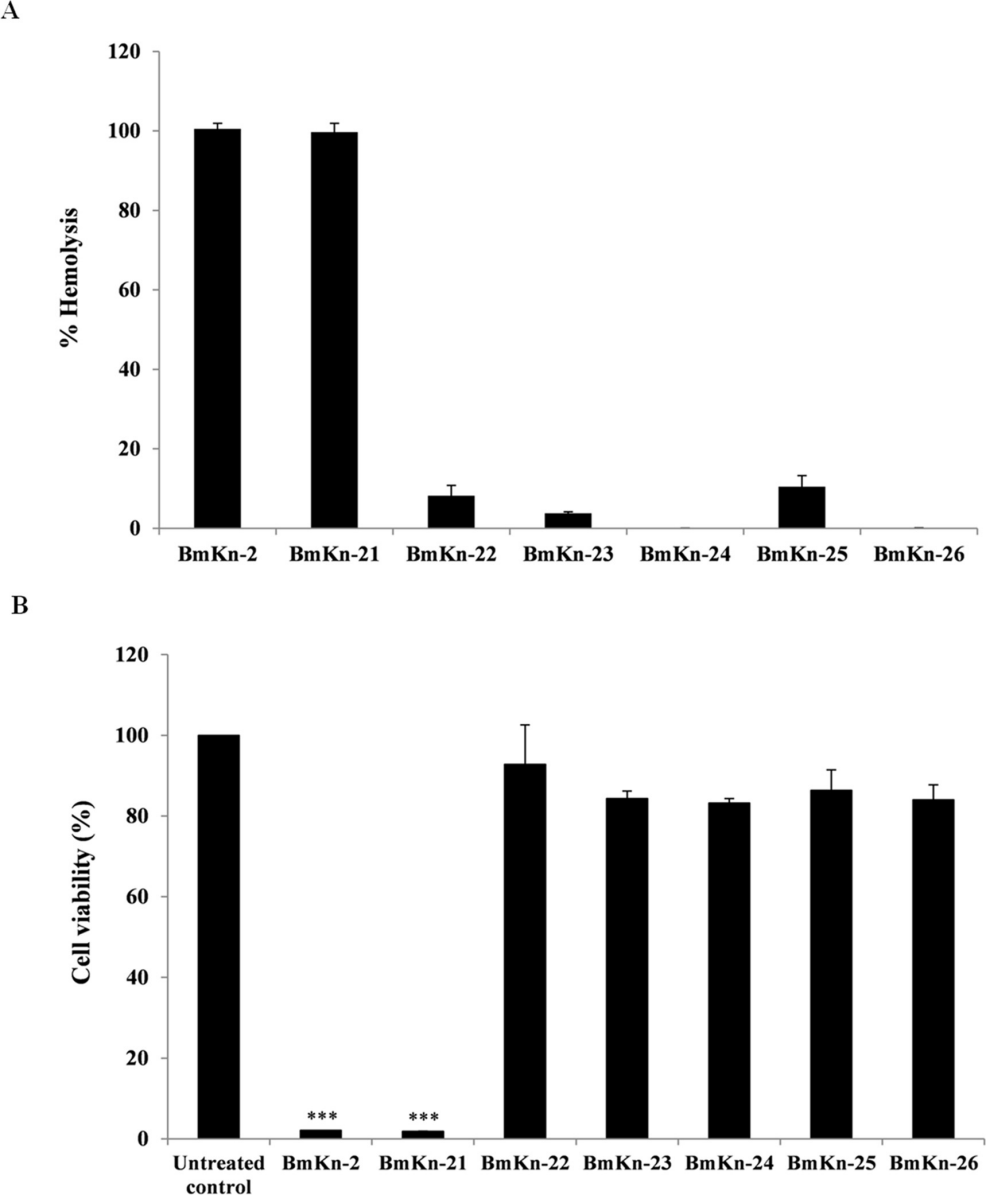
Toxicity of BmKn‒2 peptide and its derivatives against mammalian cells. Hemolytic activity of BmKn‒2 peptide and derivatives against sheep red blood cells (A), toxicity of BmKn‒2 peptide and derivatives against L929 cells determined by MTT assay (B). Values are expressed as mean ± SEM of three independent experiments. ***, *P* < 0.001 compared with the untreated control.

The hemolytic activities of BmKn‒2 peptide and its derivatives against sheep red blood cells were determined as an indication of their toxicity towards mammalian cells. As presented in Fig 3A, it was found that incubation of red blood cells with BmKn‒2 and BmKn‒21 peptides resulted in complete (100%) lysis of red blood cells. In contrast, less or no hemolysis was observed with BmKn‒22, BmKn‒23, BmKn‒24, BmKn‒25 and BmKn‒26 peptides. It is also interesting to note that % lysis of red blood cells after the exposure to the BmKn‒22, BmKn‒23, BmKn‒24, BmKn‒25 and BmKn‒26 peptides was remarkably reduced as compared with the parental BmKn‒2 peptide.

To further examine the toxicity of the test peptides against mammalian cells, the MTT assay was also performed on L929 cells. As shown in Fig 3B, significant decrease (*P* < 0.001) in viability of cells treated with BmKn-2 and BmKn-21 was obviously seen, compared with the untreated control. On contrary, treatment with BmKn‒22, BmKn‒23, BmKn‒24, BmKn‒25 and BmKn‒26 peptides produced low effects on cell viability, suggesting low toxicity of these peptides.

### Dose-dependent inhibitory effects of BmKn‒22 and BmKn‒23 peptides on biofilm formation and established biofilms of *P*. *aeruginosa*

**Fig 4:**
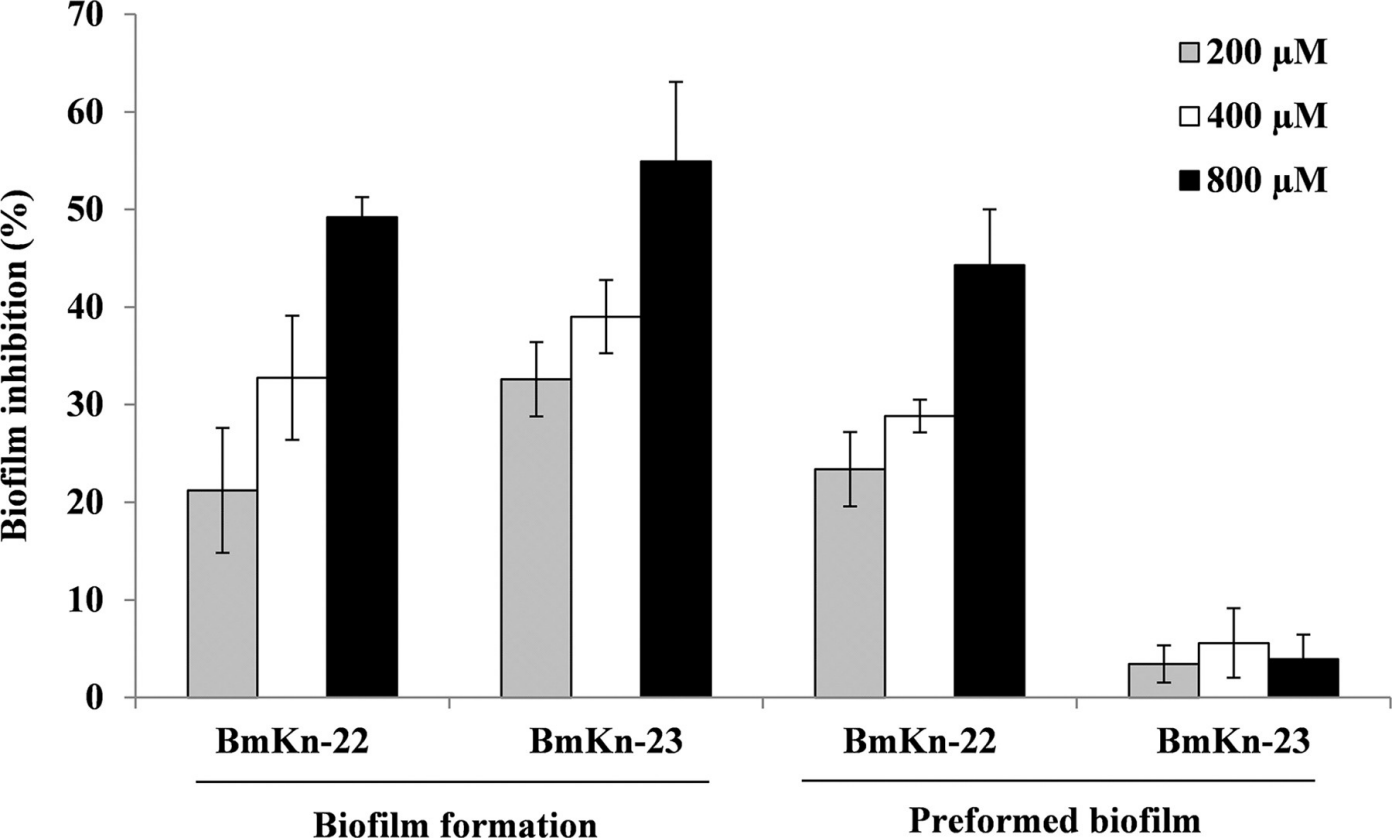
Inhibitory effects of BmKn‒22 and BmKn‒23 peptides on biofilm formation and preformed (24-h old) biofilms of *P*. *aeruginosa*. Data are represented as mean ± SEM of three independent experiments.

Through the combined results of antibiofilm activity, bacterial growth and toxicity towards mammalian cells, BmKn‒22 and BmKn‒23 peptides were selected for additional assessments for their dose-dependent inhibitory effects on biofilm formation as well as established biofilms of *P*. *aeruginosa*. As presented in Fig 4, BmKn‒22 and BmKn‒23 peptides exhibited the dose-dependent inhibitory activities against the formation of *P*. *aeruginosa* biofilms, with % inhibition ranged from 21.23‒ 49.21% and 32.60 ‒ 54.92%, respectively. The BmKn‒22 peptide also showed strong inhibitory activity against 24 h-preformed *P*. *aeruginosa* biofilms and this effect appeared to be dose-related (% inhibition ranged from 23.38 ‒ 44.31%). No eradication activity on preformed *P*. *aeruginosa* biofilms was, however, observed with BmKn‒23 peptide.

### Effect of BmKn‒22 peptide on pyocyanin production of *P*. *aeruginosa*

**Fig 5:**
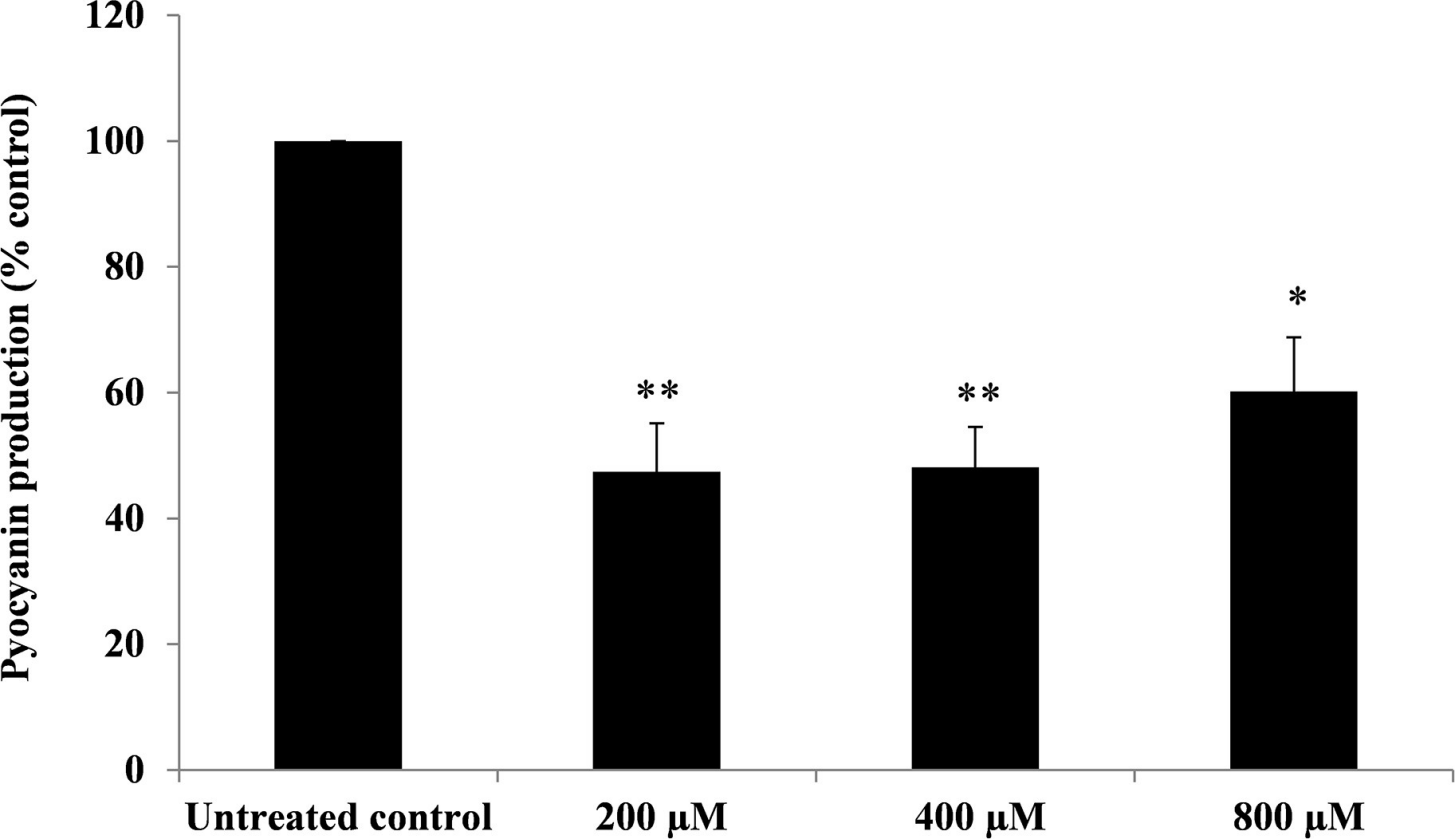
Effect of BmKn‒22 peptide on pyocyanin production of *P*. *aeruginosa*. *P*. *aeruginosa* were treated with BmKn‒22 peptide at concentrations of 200 ‒ 800 μM for 24 h, and pyocyanin was determined by chloroform extraction method. Data are expressed as mean ± SEM of three independent experiments. *, *P* < 0.05 and **, *P* < 0.01 compared with the untreated control.

Effect of the BmKn‒22 peptide on pyocyanin production of *P*. *aeruginosa* is presented in Fig 5. It was found that BmKn‒22 peptide significantly (*P* < 0.05) decreased the production of pyocyanin from *P*. *aeruginosa*, with % inhibition ranged from 39.84–52.60%.

### Effect of BmKn‒22 peptide on mRNA expression of quorum sensing-related genes in *P*. *aeruginosa*

**Fig 6:**
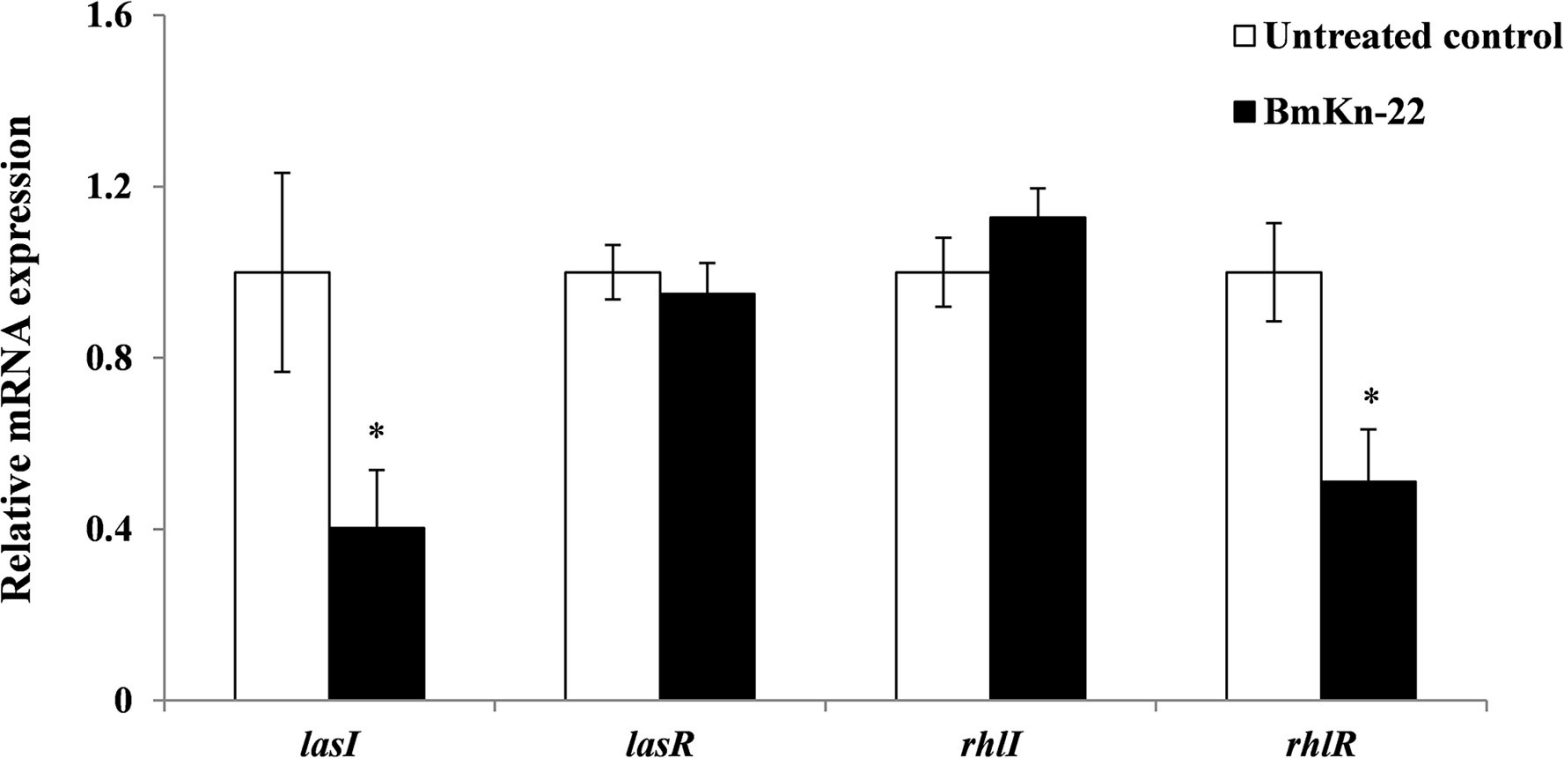
Effect of BmKn‒22 peptide on mRNA expression of quorum sensing-related genes in *P*. *aeruginosa*. Bacterial cells were treated with BmKn‒22 peptide for 10 h and the levels of mRNA expression determined by quantitative real-time PCR. Values are expressed as the mean ± SEM of two independent experiments done in quadruplicate. *, *P* < 0.05 compared with the untreated control.

To gain insight into the mechanisms by which the BmKn‒22 peptide inhibited biofilms and pyocyanin production of *P*. *aeruginosa*, the mRNA expression of the quorum sensing-related genes *lasI*, *lasR*, *rhlI* and *rhlR* was examined by quantitative real-time PCR. As shown in Fig 6, BmKn‒22 peptide significantly (*P* < 0.05) decreased the mRNA expression of *lasI* and *rhlR* genes, while no alteration in *lasR* and *rhlI* mRNA expression was detected.

### Antibiofilm activity of BmKn‒22 peptide in combination with azithromycin

**Fig 7:**
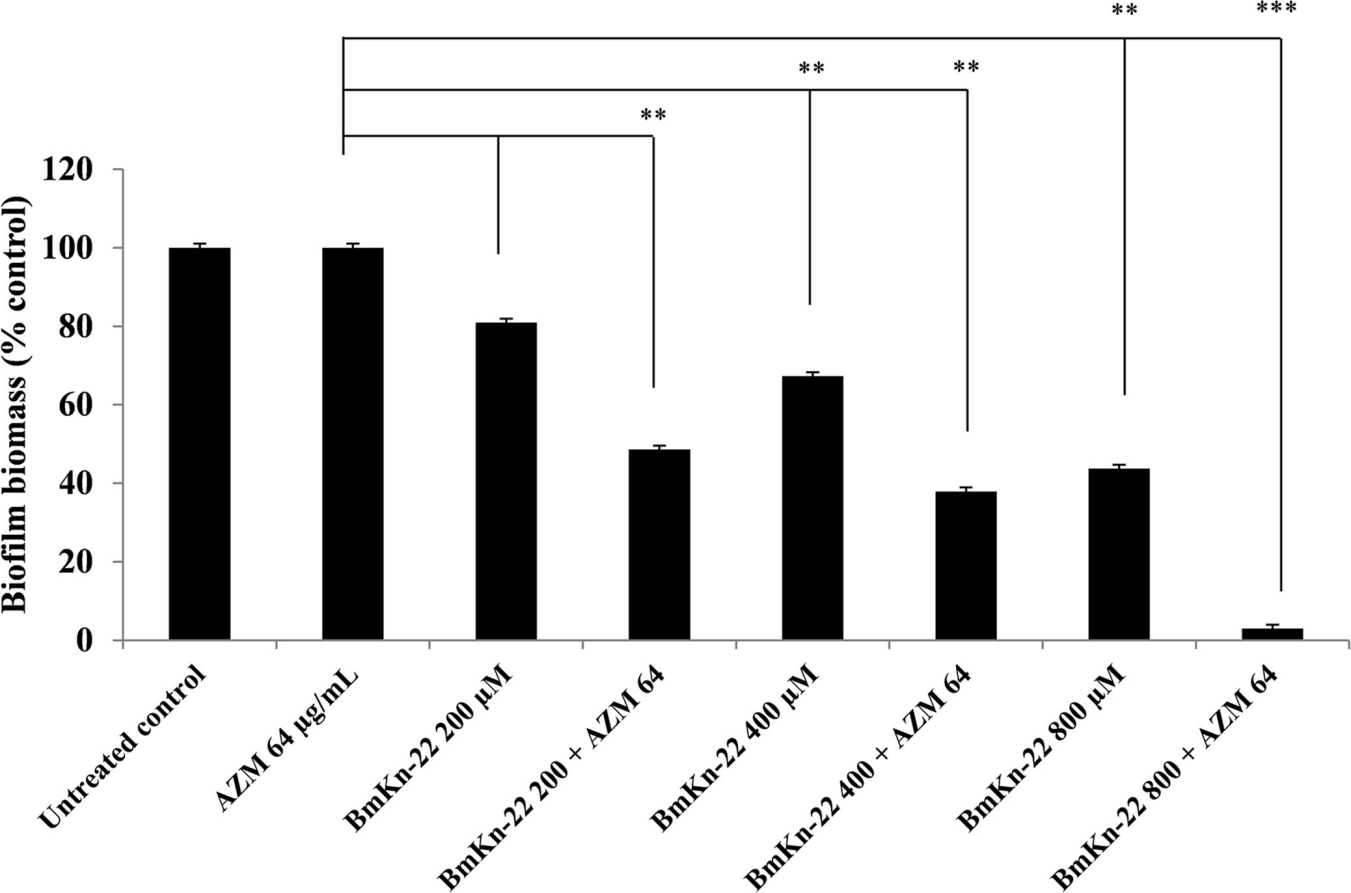
Effect of BmKn‒22 peptide and azithromycin, tested alone or in combination, on *P*. *aeruginosa* biofilms. Bacterial cells were treated with BmKn‒22 peptide alone or in combination with azithromycin for 24 h at 37°C, and biofilm biomass was assessed by crystal violet staining assay and OD measured at 550 nm. Values are expressed as the mean ± SEM of three independent experiments. **, *P* < 0.01 and ***, *P* < 0.001.

In order to determine a possible enhancement of activity when BmKn‒22 peptide was combined with the commonly used antibiotic, biofilm biomass of *P*. *aeruginosa* was examined at different concentrations of BmKn‒22 peptide in combination with sub-MIC (1/2 of MIC) dose of azithromycin (64 μg/mL). As seen in Fig 7, biofilm biomass of *P*. *aeruginosa* was considerably reduced when BmKn‒22 peptide was combined with azithromycin, compared with peptide or antibiotic alone. Up to 51.39 and 62.05% biofilm reduction was observed when BmKn‒22 peptide at 200 and 400 μM, respectively was combined with 64 μg/mL azithromycin, and increased peptide concentration to 800 μM displayed a substantial biofilm reduction activity (96.97%).

## Discussion

The emergence of multidrug resistance and the reduced effectiveness of conventional antibiotic therapy, together with the fast running out of treatment options for *P*. *aeruginosa*‒associated biofilm infections have set the priority to search for new and effective molecules against such bacterial biofilms. Using a series of the BmKn‒2 scorpion venom peptide and its derivatives, this study clearly showed that among the peptides tested, BmKn‒22 peptide displayed the most promising inhibitory activity against *P*. *aeruginosa* biofilms without affecting the bacterial growth. This peptide was not only capable of inhibiting the formation of *P*. *aeruginosa* biofilms, but also disrupting the 24-h preformed biofilms of *P*. *aeruginosa*. Our findings thus suggested that BmKn‒22 peptide was effective against both forming and established biofilms of *P*. *aeruginosa*. Additionally, BmKn‒22 peptide also exerted inhibitory activity against pyocyanin production of *P*. *aeruginosa*. Pyocynanin is a potent virulence factor of *P*. *aeruginosa* that has the ability to generate reactive oxygen species by the direct oxidation of the reduced glutathione pool of mammal cells and the concomitant reduction of oxygen [32], and are related directly to host damage. Pyocyanin also plays a significant role in promoting *P*. *aeruginosa* biofilm development which occurs via extracellular DNA release through H_2_O_2_ mediated cell lysis [33]. The ability of BmKn‒22 peptide to inhibit such a potent virulence factor therefore strengthens the powerful antibiofilm activity of this peptide. Since biofilm inhibitory activity of BmKn‒22 peptide observed in this study was not related to its growth inhibition of *P*. *aeruginosa*, this peptide may apply milder evolutionary pressure that does not favor the development of the troublesome antibiotic resistance [34]. The fact that BmKn‒22 peptide exhibited very low toxicity against mammalian cells, our observations thus indicated antibiofilm potential of BmKn‒22 and warrant further development of this peptide for treatment of *P*. *aeruginosa*‒related biofilm infections.

*P*. *aeruginosa* employed two major quorum-sensing systems, the *lasI/R* and *rhlI/R* systems, to orchestrate the production of virulence factors and to regulate the biofilm development [35]. In these systems, *lasI* and *rhlI* are involved in autoinducer synthesis, and *lasR* and *rhlR* code for transcriptional regulators [36]. When the threshold concentration of the autoinducer acylated homoserine lactones is reached, the binding to a transcriptional activator induces target virulence gene expression. Several lines of evidence have also demonstrated that the *las* system is implicated in the formation and development of biofilm [37], and regulates the expression of the *rhl* system [38]. Moreover, the *las* gene has been reported to be expressed in a large number of cells during the initial phase of biofilm development [39], and its expression remained constant throughout the infection [40]. A study carried out by Davies and colleagues [37] reported that a *P*. *aeruginosa* wild type formed structured biofilms with large mushroom-shaped structures, while the corresponding *lasI* quorum-sensing mutant formed flat and undifferentiated biofilms. The flat biofilms formed by the *lasI* mutant were susceptible to treatment with the detergent sodium dodecyl sulphate (SDS), while the structured biofilms formed by the wild type were tolerant [37]. Similar study by Allesen-Holm and colleagues [41] also found that biofilms formed by *lasIrhlI* mutant contained less extracellular DNA than biofilms formed by the wild type, and the mutant biofilms were more susceptible to treatment with SDS than the wild-type biofilm. Extracellular DNA functions as a cell-to-cell interconnecting matrix component in biofilms and is important for biofilm formation and stability. Moreover, the *lasI* and *rhlI* mutants of *P*. *aeruginosa* greatly reduced transcription of the *pel* operon, which is essential for the production of a glucose-rich matrix exopolysaccharide [42]. However, chemical complementation of the *lasI* mutant with 3-oxo-dodecanoyl homoserine lactone restores *pel* transcription to the wild-type level and biofilm formation ability [42]. Asides, the *rhl* system is required for maintaining noncolonized channels surrounding macrocolonies biofilm architecture [43] and promotes microcolonies formation, thereby facilitating three-dimensional mushroom-shaped structures formation in later stage [44]. Our study herein demonstrated that BmKn‒22 peptide significantly reduced *lasI* and *rhlR* expression, suggesting that the BmKn‒22 peptide-mediated inhibition of *P*. *aeruginosa* biofilms and virulence factors is achieved through the key components of quorum-sensing systems. Considering the central role of quorum-sensing systems in regulating biofilm formation and virulence factor production, interference of such significant systems rather than direct killing by inhibiting growth of bacteria would produce less selection pressure for the development of resistance we are currently facing. Interference of quorum-sensing has become a promising approach for the development of novel therapies to control infectious diseases, in particular those of biofilm-associated [45]. In this context, BmKn‒22 peptide would represent a promising molecule for control *P*. *aeruginosa*‒related biofilm infections.

In the present study, a series of BmKn‒2 scorpion venom peptides were assessed for their inhibitory activities against *P*. *aeruginosa* biofilms. These peptides were generated by sequentially removing amino acids from C-terminus of the parental BmKn‒2 peptide. Our results revealed that while BmKn‒2, BmKn‒21, BmKn‒22 and BmKn‒23 peptides exhibited strong antibiofilm activities, BmKn‒24, BmKn‒25 and BmKn‒26 peptides showed less pronounced inhibitory activities. Considering the amino acid sequences and biofilm inhibitory activities of these peptides, our findings suggested that “FIGAIARLLS” be the minimum amino acid sequences required for such inhibitory activities. Although substantial reduction in *P*. *aeruginosa* biofilms was observed with BmKn‒2 and BmKn‒21 peptides, complete hemolytic activity of these peptides was obviously evident. Toxicity of antimicrobial peptides towards higher eukaryotic cells has always been a major barrier that limits their clinical utility [46], thereby preventing their development as future therapeutics. Helicity and net charge of cationic antimicrobial peptides has been described to be directly correlated with hemolytic activity [47, 48]. Thus, it is likely that high percentages of helicity together with net charges of BmKn‒2 and BmKn‒21 peptides would contribute to the complete lysis of red blood cells observed in this study. Nevertheless, modification of the parental BmKn‒2 peptide by truncation of isoleucine-phenylalanine (IF) and lysine-isoleucine-phenylalanine (KIF) at C-terminal ends to obtain the respective BmKn‒22 and BmKn‒23 peptides resulted in the dramatically decreased hemolysis, implying a possible role of such amino acid residues in hemolytic activity. Strong influences of phenylalanine in the hemolytic activity have been reported by several studies [49, 50]. When tested for antibiofilm activity, both the BmKn‒22 and BmKn‒23 peptides displayed inhibition of *P*. *aeruginosa* biofilm formation. However, BmKn‒22 was the only peptide that exerted inhibitory activity against preformed biofilms of *P*. *aeruginosa*. These two peptides differed in a single amino acid residue at the C-terminal end, and BmKn‒22 having more promising activity. In this regard, the presence of lysine residue (K) in BmKn‒22 peptide, but not in BmKn‒23, may contribute to the differences in physico-chemical properties including net charge, hydrophobicity and helix, and these parameters would generate structural basis most favorable for the potent inhibitory activity. In light of our observations, the findings reported here provide valuable evidence for the successful design and development of a potent peptide against *P*. *aeruginosa* biofilms.

Combination of antibiotics with different killing mechanisms remains nowadays the best solution of the treatment of biofilm-related infections. However, high doses of antibiotics often lead to significant undesirable side effects for the patients, and repeated exposure of antibiotics can give rise to the development of multidrug resistance [51, 52]. In the present study, antibiofilm potential of BmKn‒22 peptide in combination with sub-MIC dose (1/2 MIC) of azithromycin was assessed. Azithromycin is a known antibiotic for treatment of *P*. *aeruginosa* infections and has been in use for several years [53]. It was found that combination of BmKn‒22 peptide with azithromycin resulted in the dramatic increase in the antibiofilm activity against *P*. *aeruginosa*. Combination of BmKn‒22 peptide and azithromycin also reduced dose of peptide required for antibiofilm activity. It is also interesting to note that while significant biofilm inhibition was not evident with sub-MIC dose (1/2 MIC) of azithromycin alone, remarkable inhibitory activity was obtained when combined with BmKn‒22 peptide. The observation from this present study suggested that such combination would potentiate the antibiofilm activity of azithromycin against *P*. *aeruginosa*, thereby its efficacy against *P*. *aeruginosa*‒related biofilm infections increases.

## Conclusions

To our knowledge, this study demonstrates for the first time the antibiofilm potential of the modified scorpion venom peptide, BmKn‒22 against *P*. *aeruginosa*. BmKn‒22 peptide was shown to be effective against both forming and preformed biofilms of *P*. *aeruginosa*. This peptide was also capable of inhibiting the production of virulence factor pyocyanin of *P*. *aeruginosa*. The inhibitory mechanisms involved the down-regulation of *lasI* and *rhlR*, the key components of quorum-sensing systems. Combination of BmKn‒22 peptide with antibiotic azithromycin led to a remarkable reduction *P*. *aeruginosa* biofilms. Since this peptide exhibited very low toxicity, all our results therefore indicate that the BmKn‒22 peptide is a potential antibiofilm agent against *P*. *aeruginosa* for the development of agents against *P*. *aeruginosa*‒related biofilm infections.

## Supporting information

S1 File The primary data underlying our results. (DOCX)
