# Supplementary material for: A scorpion venom peptide derivative BmKn‒22 with potent antibiofilm activity against Pseudomonas aeruginosa
Source: PLoS One. 2019 Jun 14;14(6):e0218479. doi: 10.1371/journal.pone.0218479 (PMC6568410; doi:10.1371/journal.pone.0218479)
Supplement: S1 File — (DOCX) [file pone.0218479.s001.docx]

|  | **OD 550nm** | | | | | | |
| --- | --- | --- | --- | --- | --- | --- | --- |
| **treatment** | | **concentration** | **Exp. 1** | **Exp.2** | **Exp.3** | **x̄** | **SEM** |
| **Untreated control** | | - | 0.320 | 0.509 | 0.495 | 0.431 | 0.051 |
| **BmKn-2** | | 800 µM | 0.104 | 0.401 | 0.158 | 0.221 | 0.091 |
| **BmKn-21** | | 800 µM | 0.042 | 0.203 | 0.002 | 0.082 | 0.061 |
| **BmKn-22** | | 800 µM | 0.148 | 0.277 | 0.258 | 0.228 | 0.040 |
| **BmKn-23** | | 800 µM | 0.153 | 0.302 | 0.314 | 0.256 | 0.051 |
| **BmKn-24** | | 800 µM | 0.233 | 0.359 | 0.260 | 0.284 | 0.038 |
| **BmKn-25** | | 800 µM | 0.191 | 0.421 | 0.414 | 0.342 | 0.075 |
| **BmKn-26** | | 800 µM | 0.214 | 0.350 | 0.338 | 0.301 | 0.043 |

**Figure 1. Antibiofilm activities of BmKn-2 peptide and its derivatives against *P. aeruginosa***

**Figure 2. Effect of BmKn-2 peptide and its derivatives on *P. aeruginosa* growth**

|  | **Log_10_ CFU/mL** | | | | | | |
| --- | --- | --- | --- | --- | --- | --- | --- |
| **treatment** | | **concentration** | **Exp. 1** | **Exp. 2** | **Exp. 3** | **x̄** | **SEM** |
| **Untreated control** | | - | 9.082 | 9.198 | 9.268 | 9.183 | 0.054 |
| **BmKn-2** | | 800 µM | 6.755 | 6.448 | 7.411 | 6.872 | 0.283 |
| **BmKn-21** | | 800 µM | 6.000 | 6.314 | 6.236 | 6.183 | 0.094 |
| **BmKn-22** | | 800 µM | 9.235 | 9.292 | 9.443 | 9.323 | 0.061 |
| **BmKn-23** | | 800 µM | 9.488 | 9.359 | 9.440 | 9.429 | 0.037 |
| **BmKn-24** | | 800 µM | 9.304 | 9.273 | 9.364 | 9.313 | 0.026 |
| **BmKn-25** | | 800 µM | 9.255 | 9.288 | 9.335 | 9.293 | 0.023 |
| **BmKn-26** | | 800 µM | 9.213 | 9.213 | 9.314 | 9.247 | 0.033 |

**Figure 3. Toxicity of BmKn‒2 peptide and its derivatives against mammalian cells**

**Figure 3A. Hemolytic activity of BmKn‒2 peptide and derivatives against sheep red**

**blood cells**

|  | **% Hemolysis** | | | | | | |
| --- | --- | --- | --- | --- | --- | --- | --- |
| **treatment** | | **concentration** | **Exp. 1** | **Exp.2** | **Exp. 3** | **x̄** | **SEM** |
| **BmKn-2** | | 800 µM | 103.330 | 98.962 | 99.198 | 100.497 | 1.418 |
| **BmKn-21** | | 800 µM | 103.086 | 98.672 | 97.275 | 99.678 | 2.207 |
| **BmKn-22** | | 800 µM | 14.906 | 9.709 | 0.000 | 8.205 | 2.598 |
| **BmKn-23** | | 800 µM | 5.239 | 6.058 | 0.000 | 3.765 | 0.409 |
| **BmKn-24** | | 800 µM | 0.040 | 0.000 | 0.000 | 0.013 | 0.020 |
| **BmKn-25** | | 800 µM | 9.138 | 14.647 | 7.692 | 10.492 | 2.754 |
| **BmKn-26** | | 800 µM | 0.000 | 0.124 | 0.106 | 0.077 | 0.062 |

**Figure 3B. Toxicity of BmKn‒2 peptide and derivatives against L929 cells determined**

**by MTT assay**

|  | **Cell viability (%)** | | | | | | |
| --- | --- | --- | --- | --- | --- | --- | --- |
| **treatment** | | **concentration** | **Exp. 1** | **Exp.2** | **Exp. 3** | **x̄** | **SEM** |
| **Untreated control** | | **-** | 100.000 | 100.000 | 100.000 | 100.000 | 0.000 |
| **BmKn-2** | | 800 µM | 2.236 | 2.027 | 1.988 | 2.083 | 0.019 |
| **BmKn-21** | | 800 µM | 1.974 | 1.699 | 1.835 | 1.836 | 0.067 |
| **BmKn-22** | | 800 µM | 108.407 | 75.218 | 94.787 | 92.804 | 9.784 |
| **BmKn-23** | | 800 µM | 80.383 | 84.400 | 88.135 | 84.306 | 1.867 |
| **BmKn-24** | | 800 µM | 84.274 | 81.538 | 83.751 | 83.188 | 1.106 |
| **BmKn-25** | | 800 µM | 82.909 | 93.165 | 83.012 | 86.362 | 5.076 |
| **BmKn-26** | | 800 µM | 85.233 | 87.113 | 79.724 | 84.023 | 3.694 |

**Figure 4.** **Inhibitory effects of BmKn-22 and BmKn-23 peptides on biofilm formation and preformed (24-h old) biofilms of *P. aeruginosa***

|  | | **Biofilm inhibition (%)** | | | | | | | |
| --- | --- | --- | --- | --- | --- | --- | --- | --- | --- |
|  | | **Biofilm formation** | | | | | | | |
| **treatment** | **concentration** | | **Exp. 1** | **Exp. 2** | **Exp. 3** | **x̄** | | **SEM** | |
|  | 200 µM | | 27.575 | 27.680 | 8.419 | 21.225 | | 6.402 | |
| **BmKn-22** | 400 µM | | 20.197 | 40.825 | 37.257 | 32.760 | | 6.365 | |
|  | 800 µM | | 46.468 | 53.212 | 47.945 | 49.208 | | 2.046 | |
|  | 200 µM | | 28.654 | 28.944 | 40.209 | 32.603 | | 3.804 | |
| **BmKn-23** | 400 µM | | 34.053 | 36.612 | 46.402 | 39.022 | | 3.763 | |
|  | 800 µM | | 44.669 | 49.083 | 70.992 | 54.915 | | 8.139 | |
|  | | **Preformed biofilm** | | | | | | | |
| **peptide** | **concentration** | | **Exp. 1** | **Exp. 2** | **Exp. 3** |  | **x̄** | | **SEM** |
|  | 200 µM | | 30.458 | 17.360 | 22.328 |  | 23.382 | | 3.817 |
| **BmKn-22** | 400 µM | | 27.005 | 32.202 | 27.300 |  | 28.835 | | 1.685 |
|  | 800 µM | | 44.831 | 34.141 | 53.943 |  | 44.305 | | 5.722 |
|  | 200 µM | | 0.000 | 3.802 | 6.512 |  | 3.438 | | 1.888 |
| **BmKn-23** | 400 µM | | 12.191 | 4.570 | 0.000 |  | 5.587 | | 3.555 |
|  | 800 µM | | 3.155 | 0.000 | 8.639 |  | 3.931 | | 2.523 |

**Figure 5. Effect of BmKn-22 peptide** **on pyocyanin production of *P. aeruginosa***

| **Pyocyanin production (% control)** | | | | | | |
| --- | --- | --- | --- | --- | --- | --- |
| **treatment** | **concentration** | **Exp. 1** | **Exp. 2** | **Exp. 3** | **x̄** | **SEM** |
| **Untreated control** | **-** | 100.000 | 100.000 | 100.000 | 100.000 | 0.000 |
| **BmKn-22** | 200 µM | 62.849 | 40.419 | 38.918 | 47.395 | 7.738 |
|  | 400 µM | 59.310 | 37.273 | 47.889 | 48.157 | 6.363 |
|  | 800 µM | 68.994 | 42.898 | 68.601 | 60.164 | 8.634 |

**Figure 6. Effect of BmKn-22 peptide** **on mRNA expression of quorum sensing ‒ related genes in *P. aeruginosa***

| **Relative mRNA expression** | | | | | |
| --- | --- | --- | --- | --- | --- |
| ***lasI*** | | | | | |
| **treatment** | **concentration** | **Exp. 1** | **Exp. 2** | **x̄** | **SEM** |
| **Untreated control** | - | 1.000 | 1.000 | 1.00 | 0.232 |
| **BmKn-22** | 800 µM | 0.314 | 0.491 | 0.403 | 0.135 |
| ***lasR*** | | | | | |
| **treatment** | **concentration** | **Exp. 1** | **Exp. 2** | **x̄** | **SEM** |
| **Untreated control** | - | 1.000 | 1.000 | 1.000 | 0.063 |
| **BmKn-22** | 800 µM | 0.732 | 1.166 | 0.949 | 0.072 |
| ***rhlI*** | | | | | |
| **treatment** | **concentration** | **Exp. 1** | **Exp. 2** | **x̄** | **SEM** |
| **Untreated control** | - | 1.000 | 1.000 | 1.000 | 0.080 |
| **BmKn-22** | 800 µM | 0.929 | 1.325 | 1.127 | 0.068 |
| ***rhlR*** | | | | | |
| **treatment** | **concentration** | **Exp. 1** | **Exp. 2** | **x̄** | **SEM** |
| **Untreated control** | - | 1.000 | 1.000 | 1.000 | 0.114 |
| **BmKn-22** | 800 µM | 0.408 | 0.614 | 0.511 | 0.122 |

**Figure 7. Effect of BmKn-22 peptide** **and azithromycin, tested alone or in combination, on *P. aeruginosa* biofilms.**

|  | **Biofilm biomass (% control)** | | | | | | |
| --- | --- | --- | --- | --- | --- | --- | --- |
| **treatment** | | **concentration** | **Exp. 1** | **Exp.2** | **Exp.3** | **x̄** | **SEM** |
| **Untreated control** | | - | 100.000 | 100.000 | 100.000 | 100.000 | 0.000 |
| **AZM** | | 64 µg/ml | 100.000 | 100.000 | 100.000 | 100.000 | 0.000 |
|  | | 200 µM | 94.033 | 82.424 | 66.251 | 80.903 | 5.804 |
| **BmKn-22** | | 400 µM | 75.858 | 68.773 | 57.297 | 67.309 | 3.542 |
|  | | 800 µM | 51.716 | 46.698 | 32.845 | 43.753 | 2.509 |
| **BmKn-22 200+AZM64** | | - | 61.392 | 43.402 | 41.041 | 48.612 | 8.994 |
| **BmKn-22 400+AZM64** | | - | 22.372 | 47.641 | 43.843 | 37.952 | 12.634 |
| **BmKn-22 800+AZM64** | | - | 2.393 | 6.698 | 0 | 3.030 | 2.152 |
